# Analysis of color stability and degree of conversion of different types of resin composites

**DOI:** 10.1590/1807-3107bor-2024.vol38.0003

**Published:** 2024-01-05

**Authors:** Mylena Proença COSTA, Juliana Carvalho JACOMINE, Victor MOSQUIM, Daniella Cristo SANTIN, Giovanna Speranza ZABEU, Maria Angélica Silvério AGULHARI, Rafael Francisco Lia MONDELLI, Heitor Marques HONÓRIO, Linda WANG

**Affiliations:** (a) Department of Operative Dentistry, Endodontics and Dental Materials, Bauru School of Dentistry, University of São Paulo, Bauru, SP, Brazil.; (b) Department of Pediatric Dentistry, Orthodontics and Public Health, Bauru School of Dentistry, University of São Paulo, Bauru, SP, Brazil.

**Keywords:** Color, Composite Resins, Dental Restoration Failure, Materials Testing, Polymerization

## Abstract

Resin composites containing surface pre-reacted glass (S-PRG) have been introduced to reduce demineralization and improve remineralization of the tooth structure. However, water diffusion within the material is necessary for its action, which can impair its overall physicomechanical properties over time, including color stability. This study aimed to evaluate the color stability and related degree of conversion (DC) of four resin composites. Discs (6 x 4 mm, n = 5/group) of microhybrid (MH), nanofilled (NF), nanohybrid (NH), and S-PRG-based nanohybrid (S-PRG-NH) composites with two opacities (A2/A2E and A2O/A2D) were prepared. Color (CIELab and CIEDE2000) was evaluated with a spectrophotometer after aging in grape juice (2 x 10 min/10mL/7days). The DC was analyzed by using Fourier transform infrared spectroscopy before and after light-curing. Data were statistically analyzed by using two-way analysis of variance and post-hoc least significant difference tests (p<0.05). In the color stability analysis, the interaction between filler type and opacity was significant (CIELab, p = 0.0015; CIEDE2000, p = 0.0026). NH presented the highest color stability, which did not differ from that of MH. The greatest color alteration was observed for S-PRG-NH. S-PRG fillers also influenced DC (p < 0.05). The nanohybrid resin composite presented favorable overall performance, which is likely related to its more stable organic content. Notwithstanding the benefits of using S-PRG-based nanohybrid resins, mostly in aesthetic procedures, professionals should consider the susceptibility of such resins to color alteration, probably due to the water-based bioactive mechanism of action.

## Introduction

Resin composites are often indicated to replace lost tooth structures because of their ability to mimic the shape and color of natural teeth, resulting in a nearly imperceptible restoration.^1^ In addition, physical and chemical properties allow resin-based restoration to endure daily oral challenges and preserve its integrity, luster, and color stability over the years.^2^However, there is no single ideal material for all clinical situations; therefore, it is necessary to know the characteristics and behaviors of each material for better-informed decisions.^3-5^

As established in the literature, the composition of materials,^6^filler size, and their distribution modulate the interaction of a resin composite with light^7^ by having a direct impact on the material roughness.^3^ Nanosized composites have shown reduced surface roughness when compared to their macro and microsized counterparts.^8^ This is important because increased surface roughness may lead to increased staining,^6^ particularly in patients with frequent intake of staining beverages, such as coffee,^9^ wine,^10^ and grape juice,^11^ whose low pH can also harm the integrity of the surface.^12^ Thus, to improve the performance of resin composites over time, the industry has been implementing modifications in the concentration, amount, type, and size of fillers.^13-15^

The optical properties of resin composites have been a significant factor, as their interaction with light plays an important role in mimicking tooth structures. Therefore, their opacity and/or translucency has enhanced layering techniques, enabling a higher level of reproducibility of natural dental aspects. The level of translucency of these materials is also mainly regulated by their composition and configuration of inorganic fillers.^16^ Most resin composites on the market are available in different opacities generally referred to as dentin (opaque) and enamel (translucent) resins. The variation in composition, as well as the optical interaction with light and pigments, can influence the color stability of these materials.

Additionally, another major concern with resin composite restorations is the frequent occurrence of dental caries around restorations. To overcome this clinical problem, ion-releasing composites have been developed, such as bioactive glass composites or xerogel-based multionic systems, including surface pre-reacted glass (S-PRG) fillers.^17-20^ These fillers consist of a pre-reacted fluorosilicate filler with a polyacrylic acid-treated surface layer. They combine core particles containing glass ionomer in a resin matrix.^14,18,20^As a consequence of the release of fluoride, sodium, boron, aluminum, silicate, and strontium, these resins can reduce demineralization and improve remineralization.^18-22^In addition, because of the release of boron and fluoride, they have antibacterial properties, thereby reducing the risk of carious lesions.^18,20,21^

Composites containing S-PRG fillers have great structural strength and good physical, chemical, and mechanical properties,^18,20,23^and a beyond-acceptable degree of conversion (DC). However, owing to the presence of S-PRG fillers, a certain amount of water should be diffused within the material to allow ion release into the environment,^19,24,25^ which could influence the optical properties of this resin composite over time, including color stability.^24,26^

To allow the incorporation of bioactive materials and as an attempt to address other drawbacks, the organic matrix of resin composites has gone through a lot of interesting changes in recent years.^5,27-29^ High- and low-molecular-weight monomers are therefore balanced to safeguard inorganic fillers, preserving other important clinical features, such as viscosity and DC.^30^ Special attention should be given to the DC of resin composites, as inadequate or insufficient conversion of monomers jeopardizes the mechanical properties and favors staining due to the presence of unreacted residual monomers.^31-33^Considering that S-PRG fillers partially depend on the aqueous environment, it is still unclear how their presence can interfere in color appearance.^3,34,35^

Given that unsatisfactory esthetic appearance has been considered the main reason for the replacement of anterior restorations^36^and the inclusion of ion-releasing fillers requires some level of water diffusion within the material,^37^ the optical properties of different composite resins should be further investigated. Therefore, this study aimed to evaluate the color stability and related degree of conversion (DC) of four resin composites (microhybrid, nanofilled, nanohybrid, and S-PRG-based nanohybrid) in two different opacities (A2/A2E and A2O/A2D). The first null hypothesis stated that no difference in color stability would be detected among the types of resin composites evaluated, regarding their type and opacity. The second null hypothesis stated that there was no difference in DC among the tested resins.

## Methodology

### Experimental design

This in vitro study analyzed two factors: a) resin composites classified into four levels according to the size and type of filler particles: microhybrid (MH), nanofilled (NF), nanohybrid (NH), and nanohybrid with S-PRG (S-PRG-NH); and b) opacity at two levels: translucent/enamel (T/E) and dentin/opaque (D/O). A2 color was set for all specimens. For color assessments, two response variables were used: color alteration measured by a spectrophotometer and calculated based on the CIELab (ΔE^*^_ab_) and CIEDE2000 (ΔE_00_) equations after aging in grape juice. For the DC, the absorbance of the materials before and after light-curing by Fourier transform infrared spectroscopy (FTIR) was the response variable. The experimental unit consisted of a specimen of resin composite.

The technical specifications of each material are displayed in Table 1.

**Table 1:** Classification of resin composite materials based on type of particles, commercial name, composition, opacity, and light-curing time.

Classification	Commercial name	Composition	Opacity	Light-curing	% Filled
Microhybrid	Gradia Direct – GC	UDMA, DMA, silica pre-polymers, silicon dioxide, fumed silica, silica glass, fluorine aluminum silicate glass.	A2	10 s	73.0wt%
			A2O	20 s	
Nanofilled	Filtek Z350 - 3M ESPE	BisGMA, BisEMA, TEGDMA, silane-treated ceramic, silane-treated silica, silane-treated zirconia oxide, polyethylene glycol diethanedimethacrylate, BHT and pigments.	A2E	20 s	78.5wt%
			A2D	20 s	
Nanohybrid	Spectra Smart – Dentsply	Glass powder, silica, colloidal hydrophobe, DMA, benzophenone III, EDAB (photoiniciator), concentrate FluBlau, camphorquinone, BHT butylated hydroxytoluene, yellow iron oxide, red iron oxide, black iron oxide and titanium dioxide	A2	20 s	Information not disclosed by the manufacturer
			A2O	20 s	
Nanohybrid	Beautifil II – Shofu	BisGMA, TEGDMA and Giomer technology (pre-activated glass particle with fluorine, strontium, sodium, boron, aluminum and silicate ions)	A2	10 s	83.3 wt%
(S-PRG)			A2O	10 s	

*BisGMA: Bis-phenol A di-Glycidylmethacrylate, UDMA: Urethanedimethacrylate, TEGDMA: Triethyleneglycoldimethacrylate, BisEMA: Ethoxylatebisphenol A dimethacrylate, EDAB: dimethylaminoethylbenzoate, BHT: 2,6-di-tert-butyl-p-cresol, DMA: dimethacrylate.

### Sample size calculation

In the color assessments, the effect size for the CIELab and CIEDE2000 equations was estimated to be 0.779 and 0.741, respectively, based on the findings from a pilot study. Consequently, the total sample size was estimated to be n = 4/group and n = 5/group. However, considering that the same specimens were used to calculate the color alteration using both equations, the final sample size used was n = 5/group.

For the DC analysis, the effect size was estimated to be 0.791; resulting in a total sample size of n = 4/group. However, to account for potential losses, a sample size of n=6/group was selected to be used in this study.

All sample size calculations were performed using the G*Power 3.1 software (Aichach, Germany) considering an α=0.05 and power (1-β) = 0.8.

### Specimen preparation

Forty discs (6 mm in diameter and 2 mm in thickness) were prepared and randomized into eight groups (n = 5/group) by a blinded trained operator. The material was inserted into a Teflon mold in increments of 2 mm, covered with a polyester strip, and pressed against glass plates to prevent the formation of air bubbles and to remove excess material. The upper glass plate was removed, and the resin composite was light-cured using a light-curing unit (Radii-Cal LED, 1,000mW/cm^2^; SDI, Bayswater, Victoria, Australia). The intensity was monitored with a radiometer (Demetron; Kerr, Middleton, WI, USA) for each of the five specimens. The bottom surfaces of the discs were marked with a scalpel blade, stored in deionized water for 24 hours, and protected from light at 37°C.

The top surface was polished with sequential aluminum oxide discs (Sof-lex, Pop-on, 3M ESPE, St. Paul, USA): medium (10 s), fine (10 s), and extra fine grit sizes (10 s). After polishing, the specimens were placed in an ultrasonic bath for 5 minutes for elimination of debris.

The tests were performed in accordance with the ISO guidelines 4049.^38^

### Cycling protocol

This study followed the protocol proposed by Svizero et al.^11^The artificial aging process was performed with two daily immersion cycles of 10 minutes in 10 mL of undiluted grape juice (Suco de Uva Integral UniSabor, Indústria do Sucos 4 Léguas, Caxias do Sul, RS, Brazil) for 7 days. The grape juice had a pH of 3.47 and was kept at room temperature (23 ± 2ºC). After each immersion, the specimens were washed and stored in deionized water at 37ºC. The juice and water were replaced in each cycle. After 7 days, the specimens were subjected to an ultrasonic bath with deionized water for 5 minutes, dried with absorbent paper, and their final color was measured.^11^

### Colorimetric assessment by CIELab and CIEDE2000 systems

Color was assessed on the top surface of the specimens using a spectrophotometer (Easy Shade Advance Vita; Vita Zahnfabrik, Bad Säckingen, Baden-Württemberg, Germany) on a flat matte white standardized acrylic background under standardized lighting. Three measurements for each specimen were performed by a single blinded operator, and their average was calculated. Color readings were conducted at two different time points: after polishing (at 24 hours; Δ_baseline_) and after aging in grape juice (at 7 days; Δ_final_).The spectrophotometer was calibrated for each specimen.

In CIELab (Equation 1) and CIEDE2000 (Equation 2), color systems used are based on three main parameters: L* refers to luminosity (L* = 0 = black; L* = 100 = white), a* indicates the chroma on the red-green axis (a* > 0 = red and a* < 0 = green), and b* the chroma on the yellow-blue axis (b* > 0 = yellow; b* < 0 = blue). CIEDE2000 sought to enhance the blue and gray color performance with a more specific equation, where ΔL’, AC’ and AH’ represent luminosity, chroma, and hue, respectively. ΔR = RT (ΔC’ x ΔH’) refers to the interaction between chroma and hue in the blue region. SL, SC, and SH are weighting functions that adjust the total color difference in L*, a*, and b* coordinates. KL, KC, and KH are parametric factors that serve as correction terms for experimental conditions.^39^


$$\Delta E_{ab}^{*}={\left[{\left(\Delta L^{*}\right)}^{2}+{\left(\Delta a^{*}\right)}^{2}+{\left(\Delta b^{*}\right)}^{2}\right]}^{1/2}$$

**Equation 1:** CIELab


$$\Delta E_{00}=[{\left(\frac{\Delta L^{\prime }}{K_{L}S_{L}}\right)}^{2}+{\left(\frac{\Delta C^{\prime }}{K_{C}S_{C}}\right)}^{2}+{\left(\frac{\Delta H^{\prime }}{K_{H}S_{H}}\right)}^{2}+RT\left(\frac{\Delta C^{\prime }}{K_{C}S_{C}}\right){\left(\frac{\Delta H^{\prime }}{K_{H}S_{H}}\right)}^{1/2}$$

**Equation 2:** CIEDE2000

The 50:50 acceptability and perceptibility thresholds were adopted according to Ghinea et al..^40^ The ΔE*_ab_ values corresponding to 50% acceptability and perceptibility were 3.46 and 1.80, respectively. The ΔE_00_ values corresponding to 50% acceptability and perceptibility were 2.25 and 1.30, respectively.

### Degree of conversion

The DC analysis was measured using FTIR (IRPrestige-21, Shimadzu,Tokyo, Japan) associated with an attenuated total reflectance (ATR) device.^40^Forty-eight discs were prepared (n = 6/group). For the initial reading of the unpolymerized material, the resin composite was inserted into the same Teflon mold (6 mm × 2 mm) on the ATR crystal.

All materials were light-cured (Radii-Call LED, SDI, Bayswater, Victoria, Australia) at 1,000 mW/cm^2^, according to the manufacturers’ instructions (Table 1). The final reading (polymerized material) was conducted 3 minutes after light-curing.

The readings were conducted in the absorption mode within the spectral range of 4000 to 650 cm^-1^ and included 32 scans at a resolution of 4 cm^-1^. DC was calculated based on changes in the intensity of aliphatic (1636 cm^-1^/1638 cm^-1^) and aromatic bonds (1608 cm^-1^ for NF and NH, and 1715 cm^-1^ for MH and S-PRG-NH), according to Equation 3.


$$DC=\left(1-\frac{R\text{ cured }}{R\text{ uncured }}\right)\times 100$$

**Equation 3:** Degree of conversion

### Statistical analysis

Data were entered into Microsoft Excel spreadsheets (Excel 2016; Microsoft, Redmond, USA) and analyzed for normal distribution and homogeneity using Sigma Plot software (Systat Software, Inc., San Jose, USA).

For color alteration, the data were subjected to two-way analysis of variance (ANOVA) and post-hoc least significant difference (LSD) tests. Two-way ANOVA and post-hoc LSD tests were applied for DC analysis. The significance level was set at 5% for all tests.

## RESULTS

### Colorimetric assessment by CIELab and CIEDE2000 systems

Both homogeneity (p = 0.164 for CIELab and p = 0.135 for CIEDE2000) and normality (p = 0.370 for CIELab and p = 0.656 for CIEDE2000) of the data were tested. Statistically significant differences were found for the type of fillers (CIELab and CIEDE2000, p = 0.0001) and an interaction between the type of filler and opacity (CIELab, p = 0.0015; CIEDE2000, p =0 .0026) was observed. No significant difference was found for the opacity factor (CIELab, p = 0.348; CIEDE2000, p = 0.0645). The mean ΔE values for the CIELab and CIEDE2000 systems are presented in Table 2. Table 3 shows the mean value of each analyzed parameter in a descriptive way.

**Table 2:** Means and standard deviations of ΔE values of resin composites analyzed by the CIELab and CIEDE2000 equations.

Resin composites	T/E	D/O
CIELab		
Microhybrid	3.64 (1.30) b	3.64 (0.58) b
Nanofilled	7.24 (0.99) d	5.09 (0.44) c
Nanohybrid	2.63 (0.44) ab	2.47 (0.72) a
Nanohybrid (S-PRG)	8.24 (0.86) d	9.49 (1.22) e
CIEDE2000		
Microhybrid	2.39 (0.86) bc	2.11 (0.40) b
Nanofilled	4.62 (0.58) d	3.14 (0.32) c
Nanohybrid	1.65 (0.27) ab	1.27 (0.48) a
Nanohybrid (S-PRG)	5.43 (0.65) e	6.15 (0.84) e

n = 5/group. T/E: translucent/enamel; D/O: dentin/opaque. Lowercase letters indicate statistical difference between type of materials and opacities.

**Table 3:** Descriptive means of color coordinate values for the analyzed resin composites.

Varialble	T/E					D/O				

	ΔL	ΔC	ΔH	Δa	Δb	ΔL	ΔC	ΔH	Δa	Δb
MH	-3.40	-1.15	-0.45	0.00	-1.18	-3.02	-1.84	-0.05	-0.28	-1.83
NF	-6.13	-3.69	2.51	-1.07	-3.68	-4.27	-2.94	0.77	-0.89	-2.76
NH	-2.43	-0.92	-0.51	0.16	-0.94	-1.69	-1.71	-0.25	-0.05	-1.71
S-PRG-NH	-7.71	-2.35	0.47	-0.52	-2.29	-9.07	-2.98	0.86	-0.57	-2.70

MH: microhybrid; NF: nanofilled; NH: nanohybrid; NH-S-PRG: nanohybrid (S-PRG); T/E: translucent/enamel; D/O: dentin/opaque.

Based on the CIELab equation, among the T/E resin composites, NH presented the lowest ΔE*_ab_, which did not differ from that of MH. The greatest ΔE*_ab_ was observed for S-PRG-NH, which did not differ from NF; however, both differed from MH and NH.

For the D/O resin composites, all groups were different. The lowest ΔE*_ab_ was also noted for NH, which differed from MH, NF, and S-PRG-NH. The highest ΔE*_ab_ was observed for S-PRG-NH, which differed from all others.

Nonetheless, despite these results, all ΔE*_ab_ values of both T/E and D/O composites were classified as clinically notable (> 3.46), except for NH, whose ΔE*_ab_ was the only one classified as clinically perceptible (> 1.8).

Regarding the CIEDE2000 equation, favorable outcomes were also detected for T/E composites in the case of NH and MH, which did not differ between them. The more perceptible changes were attributed to S-PRG-NH, which differed from those of the other materials. NF presented intermediate values, which differed from all groups.

Among the D/O composites for CIEDE2000, a performance similar to that of CIELab was verified. NH presented the lowest ΔE_00_, which differed from those of the other materials. MH and NF presented intermediate values of ΔE_00_, differing from each other and from both NH and S-PRG-NH. The S-PRG-NH material showed the most perceptible results and differed from all materials.

According to the established thresholds, all ΔE_00_ values of T/E were classified as clinically unacceptable (> 2.25), except for NH, whose ΔE*_ab_ values were classified as clinically perceptible (> 1.3). For D/O opacity, the materials were classified as clinically unacceptable (> 2.25), except for MH and NH materials, which were within the acceptable range (< 1.3).

### Degree of conversion

The homogeneity (p = 0.002) and normality (p = 0.109) of the data were assessed. Statistically significant differences were found between the evaluated materials, opacities, and their interaction (p < 0.0001). The DC values are listed in Table 4 and representative spectra of FTIR analyses before and after light-curing are presented in Figure.

**Table 4:** Degree of conversion (%) and standard deviation of the tested groups.

Resin composites	Opacity	

	T/E	D/O
Microhybrid	43.06 (3.49) cd	44.72 (2.57) cd
Nanofilled	59.36 (4.85) a	46.47 (6.07) c
Nanohybrid	55.43 (2.85) ab	49.53 (2.65) bc
Nanohybrid (S-PRG)	43.96 (2.67) cd	39.32 (0.79) d

N=6/group. T/ E: translucent/ enamel; D/O: dentin/ opaque. Lowercase letters indicate statistical difference between type of materials and opacities.

**Figure:**
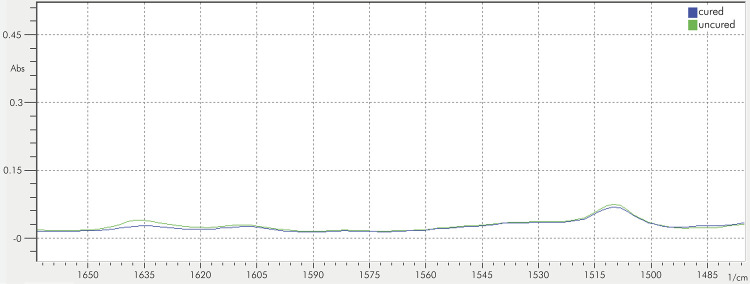
Representative FTIR spectra of a resin composite before and after light-curing.

In the case of T/E specimens, NF and NH presented higher DC compared to MH and S-PRG-NH (p < 0.05). Among O/D specimens, S-PRG-NH and MH showed a lower DC than did other materials with the same opacity (p < 0.05). Significant differences between the opacities were observed only for NF. For this material, the translucent version showed a higher DC than the opaque version (p < 0.05).

## Discussion

Discoloration of restorative materials occurs for different reasons, such as DC, titratable acidity, food colorant absorption and penetration,^1,24,32^and the size and amount of inorganic filler.^40^The tooth substrate and surface properties of the materials can also be included, as well as the type of polishing performed.^3,6,9^In the present study, for both CIELab and CIEDE2000 equations, S-PRG-NH showed the highest values for color alteration, followed by NF, MH, and NH, which presented the lowest ΔE values. Regarding opacities, NF and S-PRG-NH showed difference in terms of color stability for CIELab, which was noted only for NF in the case of CIEDE2000. Therefore, the first null hypothesis was rejected.

In clinical practice, layering of different colors and opacities of resin composites is recommended in order to achieve excellent aesthetic outcomes that closely resemble natural teeth. The overall discoloration of this material can result from extrinsic factors (e.g., pigments from food and beverages) or intrinsic factors, such as the composition of the organic matrix, size and amount of the inorganic matrix, photoinitiators, and DC.^24,40^ The opacity and translucency of these materials can also be influenced by their composition, which is mainly determined by the difference in refractive index between the organic and inorganic matrix and by the amount and size of the filler.^40^ This difference between the opacities could influence their response to discoloration; however, most materials showed no difference in color change between the two opacities (T/E and D/O) in this study, except for NF (CIELab and CIEDE2000) and S-PRG-NH (CIEDE2000).

This alteration was evident for all evaluated parameters, particularly in the case of NF-T/E compared to NF-D/O, which explains the difference of ΔE between their opacities. Although all evaluated resins showed a decrease in luminosity (ΔL), this alteration was more evident in S-PRG-NH, which was the most affected parameter. The presence of S-PRG particles in resin composites based on this technology leads to a more whitish appearance, affecting the perception of luminosity of these materials. The greater opacity of D/O group also contributed to the fact that the decrease in ΔL values became more evident for this material. In addition, both resins showed an increase in hue (ΔH) values, in contrast to MH and NH, whose values decreased. In general, the yellow-blue axis (Δb) was affected, in line with the bluish coloration of the substance used to age the specimens.

The mechanism of action of S-PRG fillers depends on water diffusion,^37^which requires higher amounts of hydrophilic monomers and higher water sorption as compared to materials not subjected to this technology.^20,24,25^Previous studies have shown a positive correlation between water sorption and staining,^24,25^ which could explain the higher staining observed for S-PRG-based composites. Nonetheless, as water is absorbed, expansion and plasticization of the resin and hydrolysis of the silane bonds occur, which can generate microcracks and the release of residual monomers.^11^ This could also favor staining and compromise the aesthetic appearance of the restoration.^28,32^ Moreover, as silane bonds are degraded, the filler particles of the resin can start to detach from the organic matrix, contributing to the increase in their staining potential due to increased roughness and pigment retention.^28,32,37^

Studies have shown that the solubility of BisGMA-based composites increases when associated with triethylene glycol dimethacrylate (TEGDMA) and decreases with urethane dimethacrylate (UDMA).^24,29^ These findings could explain the findings of the present study, in which greater staining was seen for BisGMA and TEGDMA-based composites (S-PRG-NH and NF), indicated mainly by a greater change in the ΔL* parameter,^29^ corroborating the findings of this study. The high water sorption promoted by the association of BisGMA and TEGDMA, combined with the mechanism of S-PRG fillers, could explain the staining of the materials shown in this study. The manufacturers did not specify the dimethacrylates in the tested NH and MH resins, except for the UDMA diluent used in MH. The presence of UDMA in the organic matrix could explain the higher resistance of MH to staining, but the lack of this information precludes a more robust interpretation of the results.

In this case, limitations can also be extended to other properties, and the balance of the main properties needs to be considered for the appropriate recommendation of each material.^4^

The color stability of resin composites has also been strongly associated with their DC.^31^ In this study, S-PRG-NH and MH presented the lowest DC, while NH and NF materials showed the best percentages of monomer-to-polymer conversion. A higher opacity can also influence the passage of light through the material, thus decreasing DC and leading to greater susceptibility to discoloration.^31^ The impact of this property was only observed for NF, which showed lower values for its D/O version compared to T/E. Therefore, the second null hypothesis was also rejected. Incomplete light-curing generates a greater amount of residual monomers, which are easily degraded, resulting in greater susceptibility to pigmentation.^33^

Differences in the organic matrix composition of methacrylate and the size, type, and volume of particles can affect the depth of light curing and scattering, and consequently, the DC.^30^ Hence, the volume and size of fillers in the composition of the S-PRG-based resin composite used in this study could have influenced the DC results. Ilie and Fleming^34^ compared different materials, including one with S-PRG fillers similar to this study, and the poor performance was attributed to the presence of the filler.^35^ This technology shows larger particles when compared with other technologies, and it is associated with more filler percentage, hindering light penetration into deep layers, thus decreasing the DC.^34^ S-PRG-NH presented a percentage of inorganic fillers of 83.3wt% against 73.0wt% of MH and 78.5wt% of NF. The percentage of fillers in the NH resin was not provided by the manufacturer.

Additionally, it is known that TEGDMA increases the DC,^35^ which could have contributed to the higher values observed for NF. The similar performance of S-PRG-NH, which also contains TEGDMA monomer, can be limited by these fillers, as mentioned above. Nevertheless, the presence of TEGDMA can impair the mechanical properties^35^because the monomers used in the formulation of these composites are strongly related to the staining potential and DC of the material. Future studies addressing the physicochemical properties of these resins should be conducted.

Therefore, based on the analyses in the present study, MH and NH could be used as an excellent aesthetic treatment alternative. In contrast, S-PRG-NH should be indicated in specific situations. Gordan et al. evidenced optimal performance of posterior restorations using Beautifill II.^19^ In particular, in Class II situations, this 13-year follow-up proved its clinical effectiveness. In a recent study, Toz-Akalin et al. conducted a 2-year follow-up and demonstrated how this material containing low-shrinkage organic monomers and reduced amount of S-PRG can yield more interesting results under service conditions.^5^ Therefore, as reported in other studies, clinical circumstances, such as daily brushing^10^ and polishing protocols,^12^ may also play important roles in the longevity of restorative materials. Future studies should investigate these parameters, given that repolishing can re-establish the original color of the restorative material and achieve clinically acceptable levels of aesthetic longevity.^12^

An equilibrium between the in vitro and in vivo performance of the materials promotes robust interpretation, aiding professionals to choose materials and techniques in different circumstances and assuring more precise benefits to the patients.

## Conclusions

Despite the limitations of this study, it can be concluded that the nanohybrid resin composite presented favorable overall performance, which is likely related to its more stable organic content. However, the use of S-PRG-based nanohybrid resins in aesthetic procedures should take into account their greater susceptibility to color alteration, probably due to the water-based bioactive mechanism of action. The opacity of the material may also affect the color stability and the DC of some materials.
